# New mechanism for glutamate hypothesis in epilepsy

**DOI:** 10.3389/fncel.2013.00127

**Published:** 2013-08-13

**Authors:** Chang-Hoon Cho

**Affiliations:** Epilepsy Research Laboratory, Department of Pediatrics, Abramson Research Center, Children's Hospital of PhiladelphiaPhiladelphia, PA, USA

Epilepsy is a broad range of neurological conditions that are manifested as seizures. Two major hypotheses—glutamate and potassium—have been proposed for the mechanism of epilepsy development (Fisher et al., 1976; During and Spencer, 1993). Although both hypotheses have some evidence to support, the relative contribution of potassium and glutamate to epilepsy has not been determined.

Glutamate is a major excitatory neurotransmitter in the brain and an immediate precursor for GABA in neurons and glutamine in astrocytes. Glutamate is differentially compartmentalized and metabolized via different enzymes by astrocytes and neurons and exogenous and endogenous glutamate is handled distinctively by them (McKenna, 2007). Elevated levels of glutamate have been reported in human brain tissues and animal models of epilepsy, and it is known that glutamate-induced excitotoxicity causes the neuronal death in epilepsy (Haglid et al., 1994; Coulter and Eid, 2012 for detail).

The glutamate-glutamine cycle is a major recycling mechanism of glutamate and GABA in the brain. A glutamate degrading enzyme, glutamine synthetase (GS) has been shown to be deficient in astrocytes in the epileptogenic hippocampal formation in a subset of patients with temporal lobe epilepsy (TLE) (Eid et al., 2004). This GS deficiency leads to increased glutamate levels in astrocytes as well as elevated concentrations of extracellular glutamate. Rats chronically infused with methionine sulfoximine, a GS inhibitor, showed increased glutamate in astrocytes (Perez et al., 2012).

Increased glutamate release from neurons and astrocytes and/or impaired removal of glutamate in the extra-cellular space (e.g., synaptic cleft) could raise glutamate level. Glutamate clearance is mainly performed by glutamate transporters that move glutamate and potassium across the plasma membrane (Had-Aissouni, 2012). The astroglial sodium-dependent glutamate transporter-1 (GLT-1 a.k.a. EAAT2) is the major glutamate uptake molecule in the brain, and either malfunction and/or down-regulation of GLT-1 could cause elevated glutamate level. The fact that GLT-1 null mice are epileptic supports GLT-1 involvement for increased glutamate (Tanaka et al., 1997). However, findings of GLT-1 level in animal models and human TLE are not consistent, and they do not seem to fully explain the elevated glutamate (Mathern et al., 1999; Crino et al., 2002; Proper et al., 2002; van der Hel et al., 2005). It is evident that astrocytes are potential sources of the excessive glutamate in TLE, however, the mechanism(s) for glutamate release from astrocytes has been controversial; either vesicular exocytosis or channel/transporter-mediated and whether calcium is involved or not (Tian et al., 2005).

A recent study demonstrating two different modes of glutamate release in astrocytes provides a new way of thinking for epilepsy: TREK-1, a two-pore potassium channel, can be responsible for fast glutamate release induced by GPCR (G-protein coupled receptors) activation and Bestrophin-1 (Best1), a calcium activated anion channel for slow release (Woo et al., 2012). To detect released glutamate from astrocytes, a technique called “sniffer-patch” was used: electrophysiological recording of HEK293T cells expressing a non-desensitizing mutant (GluR1-L497Y) of AMPA receptors were co-cultured with dissociated hippocampal astrocytes. An agonist peptide for PAR1 (protease-activated receptor 1) was applied to activate G-protein in astrocytes. Thus, glutamate released from astrocytes can be measured via AMPA receptor currents. Differential subcellular localization of these two channels also raises the possibility of distinct mode of their operations in epilepsy. TREK-1 is preferentially expressed at cell bodies and processes of astrocytes, while Best1 is present close to synapses. It remains to be seen whether their expression and localization in the brain is altered in epilepsy.

The involvement of TREK-1, which was identified as a potassium channel, here is particularly interesting. The potassium hypothesis of epilepsy was proposed about a half century ago (Green, 1964; Fisher et al., 1976) and inactivation mutations of several potassium channels cause human and rodent epilepsies (Benarroch, 2009). Impaired spatial potassium buffering by astrocytes will result in stronger and prolonged depolarization of glial cells and neurons in response to activity-dependent potassium release, and may thus contribute to seizure generation in this particular condition of human TLE (Hinterkeuser et al., 2000). TREK-1 null mice have increased seizure susceptibility to systemic kainate administration. Is the pore of TREK-1 permeable both to potassium and glutamate? Is there any selective mechanism of TREK-1 for one molecule over the other depending on the activating signals? The answers to these questions may aid in dissecting the relative contribution of these two molecules to hyperexcitability and epilepsy. As for Best1, the question is whether enhanced calcium signals in astrocytes directly affect Best1's function in epilepsy models (Heurteaux et al., 2004; Ding et al., 2007). Therefore, it will be very intriguing to examine the workings of TREK-1 and Best1 in animal models and human TLE.
